# Correction: Registered report: Coadministration of a tumor-penetrating peptide enhances the efficacy of cancer drugs

**DOI:** 10.7554/eLife.09169

**Published:** 2015-06-08

**Authors:** Irawati Kandela, James Chou, Kartoa Chow, Reproducibility Project: Cancer Biology

Kandela I, Chou J, Chow K, Reproducability Project: Cancer Biology. 2015. Registered report: Coadministration of a tumor-penetrating peptide enhances the efficacy of cancer drugs. *eLife* **4**:e06959. doi: 10.7554/eLife.06959

Published 22 May 2015

In the published Registered report there was an error in the iRDG peptide sequence listed in ‘Protocol 1: synthesis of iRDG’. The sequence was incorrectly listed as H-Gly-Arg-Gly-Asp-Lys-Gly-Pro-Asp-Cys-NH2. The correct sequence is: H-Cys-Arg-Gly-Asp-Lys-Gly-Pro-Asp-Cys-NH2.

This article has been corrected accordingly.

